# Trajectories and determinants of echocardiographic myocardial work indices after acute myocardial infarction: a pilot study

**DOI:** 10.1186/s44156-026-00131-5

**Published:** 2026-08-10

**Authors:** Haitham Ballo, Wail Nammas, Christian Paunonen, Jarmo Teuho, Reetta Siekkinen, Xiang-Guo Li, Anne Roivainen, Juhani Knuuti, Antti Saraste

**Affiliations:** 1 Heart Center, Turku University Hospital and University of Turku, Turku, Finland; 2 Turku PET Center, Turku University Hospital and University of Turku, Turku, Finland

**Keywords:** Myocardial work, Echocardiography, Acute myocardial infarction, Myocardial viability, Left ventricular function, Myocardial perfusion, Strain imaging

## Abstract

**Introduction:**

We evaluated the trajectories of echocardiographic myocardial work (MW) indices and their associations with left ventricle (LV) functional recovery, myocardial viability, and perfusion in patients with reperfused acute myocardial infarction (AMI).

**Methods:**

Thirty-one patients with a first ST-elevation AMI treated with percutaneous coronary intervention were prospectively enrolled. Transthoracic echocardiography and [^15^O]-water positron emission tomography (PET) were performed at a median of 7.7 ± 3.8 days post- intervention (baseline). Global and segmental absolute myocardial blood flow (MBF) were assessed using PET, and myocardial segments were graded as viable or non-viable based on perfusable tissue fraction (PTF). Echocardiographic parameters, including LV ejection fraction (LVEF), global longitudinal strain (GLS), myocardial work index (MWI), constructive work (MCW), wasted work (MWW), and work efficiency (MWE), were measured at baseline and at the 6-month follow-up. The myocardial area at risk (AAR) and the remote myocardium were defined based on the culprit coronary artery segment.

**Results:**

LVEF and GLS remained unchanged between baseline and follow-up. However, global MWI, MCW, and MWE significantly increased, while MWW decreased at the 6 months (all *p* < 0.05). An increase in MWI, but not MWE, was associated with improvement of LVEF. Neither global MWI nor MWE correlated with global MBF, but were inversely associated with peak levels of troponin T and NT-proBNP. In the AAR, regional MWI and MWE were significantly correlated with regional MBF (*r* = 0.53, *p* = 0.003, and *r* = 0.59, *p* < 0.001, respectively) and PTF (*r* = 0.41, *p* = 0.03, and *r* = 0.65, *p* < 0.001, respectively). At baseline, segmental MWI and MWE were higher in viable compared to non-viable segments. At 6 months, MWI and MWE increased in viable, but not in non-viable segments.

**Conclusions:**

Echocardiographic myocardial work indices reflect the severity of myocardial injury early after AMI and may serve as non-invasive markers of functional recovery and myocardial viability.

**Supplementary Information:**

The online version contains supplementary material available at 10.1186/s44156-026-00131-5.

## Introduction

Acute myocardial infarction (AMI) remains one of the leading causes of morbidity and mortality worldwide. It can result in left ventricular (LV) dysfunction, adverse remodelling, and eventually heart failure [1]. Post-infarction remodelling is commonly associated with larger infarct size, as indicated by elevated biomarkers such as troponin and NT-proBNP, reduced LV ejection fraction (LVEF), and increased LV volumes [2].

Speckle-tracking echocardiography enables the quantitative assessment of global and regional LV function through myocardial strain analysis. However, strain measurements are sensitive to loading conditions and may be influenced by elevated afterload [3]. To address this limitation, myocardial work (MW) indices have emerged as novel echocardiographic parameters that integrate myocardial deformation with estimated LV pressure. These indices provide a more comprehensive evaluation of myocardial performance, offering insights into contractile function, energy efficiency, and myocardial oxygen consumption. Despite their potential, data on the temporal evolution and clinical determinants of MW indices after AMI remain limited.

This study aims to evaluate the trajectories of echocardiographic MW indices over time and their associations with LV recovery, myocardial viability, and myocardial blood flow (MBF), as assessed by [^15^O] water positron emission tomography (PET), in patients with reperfused ST-elevation AMI.

## Methods

### Study cohort and design

We prospectively recruited patients who underwent primary percutaneous coronary intervention (PCI) because of the first ST-elevation AMI and had an LVEF of less than 50% during the index hospitalisation in Turku University Hospital from December 2018 to January 2021. Detailed inclusion and exclusion criteria have been published earlier [4]. Each patient signed an informed consent form. The study conforms to the Declaration of Helsinki, and the institutional review boards of the Hospital District of Southwest Finland, the Finnish Medicines Agency, and Turku University Hospital approved the study. The study was registered in clinicaltrials.gov with identifier NCT04871217.

According to the study protocol, patients underwent baseline echocardiography and [^15^O] water PET on the same day within 3 to 14 days (mean 7.7 ± 3.8 days) after AMI, and echocardiography was repeated at the 6-month follow-up (mean 210 ± 38 days after AMI). Peak cardiac troponin T and N-terminal pro-B-type natriuretic peptide (NT-pro-BNP) levels were recorded during hospitalisation and PET imaging. Data on cardiovascular risk factors, medications, and cardiovascular events were collected from electronic medical records. The myocardial area at risk (AAR) and the remote area were based on the culprit coronary arterial segment, determined from the invasive coronary angiography and electrocardiography.

### Transthoracic echocardiography

Transthoracic echocardiography was performed using Vivid E9 or E95 (GE Vingmed Ultrasound, Horten, Norway) devices with MS5 and 4Vc-D 4D probes. The echocardiographic views obtained included apical 4-chamber, 2-chamber, and long-axis views. All images were digitally stored for offline analysis (EchoPAC P C version 203; GE Vingmed, Horten, Norway). All standard measurements were performed according to the American Society of Echocardiography and European Association of Cardiovascular Imaging guidelines [5].

The LV end-diastolic volume (EDV), LV end-systolic volume (ESV), and LVEF were measured using the biplane Simpson’s method. LVEF recovery was defined as ≥5% increase in LVEF from baseline at the 6-month follow-up [6].

Myocardial global and segmental longitudinal strain were analysed using speckle-tracking echocardiography (STE). Semi-automatic software created a region of interest (ROI) covering the LV myocardium. Speckles were assumed automatically by the software, and the user confirmed proper tracking of the myocardium. In the presence of poor tracking, the observer re-adjusted the endocardial trace. Myocardial global longitudinal strain (GLS) and segmental longitudinal strain (LS) were analysed using the speckle-tracking method and reported as absolute percentages.

The quantification of MW indices was performed using the same software package as STE. It was measured by areal pressure-strain loop, obtained from non-invasive LV pressure curves with deformation acquired with STE. GLS and systolic blood pressure were synchronised by aligning the timing of the valve events (mitral and aortic valve opening and closure), confirmed by 2D evaluation in the long axis apical view. MW was evaluated from mitral valve closure to mitral valve opening. Global and segmental MW indices, including myocardial work index (MWI), constructive work (MCW), wasted work (MWW), and work efficiency (MWE) were derived from non-invasive LV pressure-strain loops [7]. In order to evaluate independent value of MW indices, these were analysed separately from LVEF, GLS and LS by a different investigator blinded to other data.

Regional myocardial work indices were calculated at the segmental level using the standard 17-segment model [8]. Segments were assigned to the area at risk and remote myocardium based on the culprit coronary artery territory, as determined by coronary angiography and electrocardiographic findings. Regional MW indices were obtained by averaging segmental values within each predefined region.

Reproducibility of myocardial work measurements was assessed in a subset of patients using intraclass correlation coefficients (ICC) (two-way mixed-effects model, absolute agreement) and coefficients of variation (CV). Both intra- and inter-observer variability were evaluated.

### Positron emission tomography

PET studies were performed using a PET/computed tomography (PET/CT) scanner (Discovery MI; GE Healthcare), as previously described [4]. In brief, [^15^O] water (Radiowater Generator; Hidex Oy) was injected as an intravenous bolus (target injected radioactivity 500 MBq) over 15 s, and dynamic PET was performed over 4 min and 40 s, starting 25 s after injection, with the patient at rest.

Images were analysed using Carimas 2.9 software (Turku PET Centre) [9]. Segmental LV myocardial blood flow (MBF) and water-perfusable tissue fraction (PTF) were quantified from [^15^O] water images as ml/min/g and g/ml, respectively, using a single-compartment model as described previously [10]. The PET measurements were displayed as polar maps. Global MBF values were corrected for a rate-pressure product using the following equation: corrected MBF = (MBF/rate-pressure product) × 10^4^ [11]. Myocardial viability was assessed at the segmental level using PTF. Segments were classified as viable when PTF was ≥ 0.60 g/mL and non-viable when PTF was < 0.60 g/mL, based on previously validated thresholds [12]

### Statistical analysis

Continuous data are reported as mean and standard deviation (SD) or median and interquartile range. The data were compared using Student’s t-test when normally distributed and with the Mann-Whitney test otherwise. Categorical data are reported as count (percentage) and compared with Chi-square or Fisher’s exact tests as Appropriate. Correlation between variables was assessed using the Pearson’s or Spearman’s rho rank correlations. Due to significant collinearity among MW indices, they were analyzed separately. Binary logistic regression was used because myocardial viability was a binary outcome (viable vs. non-viable), while MW indices were entered as continuous predictors. Odds ratio (OR) with 95% confidence interval (CI) was estimated. Receiver operating curve (ROC) analysis was performed to test the parameters associated with viability. Intra- and interobserver variability of GLS as well as intra-observer variability of MW indices were assessed using coefficient of variation and intraclass correlation coefficient in 6 randomly selected patients. Statistical analyses were done using SPSS (SPSS, Chicago, IL).

## Results

We enrolled 31 patients with the first ST-elevation AMI. Table 1 summarises their baseline characteristics. All patients underwent primary PCI at 4.9 ± 6.1 hours from symptom onset. The AAR was in the left anterior descending, right, and left circumflex coronary artery territories in 48, 29, and 23% of patients, respectively. Baseline imaging was performed at a mean of 7.7 ± 3.8 days (range 3–14 days). The distribution of imaging timing is shown in Supplementary Figure 1. No significant differences were observed in GLS, myocardial work indices, or PET-derived parameters between patients who underwent baseline echocardiography and PET ≤ 7 days (*n* = 15) and >7 days (*n* = 16) after AMI (Supplementary Table 1).

**Table 1:** Patient characteristics

Cohort	(*N* = 31)
Age (years)	64.2 ± 9.2
Male sex	28 (90.3)
Body mass index (kg/m2)	25.4 ± 4.8
Current smoking	11 (35.5)
Diabetes mellitus	3 (9.7)
Hypertension	13 (41.9)
Hypercholesterolemia	17 (54.8)
Family history of CAD	9 (31)
Time from symptoms to PCI (hours)	4.9 ± 6.1
Culprit coronary artery territory	
Left anterior descending artery	15 (48.4)
Circumflex artery	7 (22.6)
Right coronary artery	9 (29)
Post-PCI TIMI flow	
Grade 2	10 (32.3)
Grade 3	21 (67.7)
Peak troponin T (ng/L)	3884.3 ± 4391.7
Peak NT-proBNP (ng/L)	979.4 ± 871.5
Total cholesterol (mmol/L)	4.2 ± 1.1
LDL cholesterol (mmol/L)	2.9 ± 0.9
Duration of hospital stay (days)	3.7 ± 1.8
Loop diuretics	10 (32.3)
Inotropic medication	7 (22.6)

CAD, coronary artery disease; PCI, percutaneous coronary intervention; TIMI, thrombolysis in myocardial infarction; LDL, low-density lipoprotein; ACEI/ARB, angiotensin-converting enzyme inhibitor/angiotensin receptor blocker. Qualitative data are number and percentage; continuous data are mean ± SD

One patient was lost to follow-up. Consequently, 30 patients underwent baseline and the six-month follow-up echocardiography. During the follow-up period, there were no deaths or heart failure hospitalizations. One patient experienced a non-ST-elevation AMI related to a non-culprit lesion. No arrhythmias requiring device implantation were observed. There was no significant change in systolic blood pressure readings from the baseline at the six-month follow-up (126 ± 15 vs 131 ± 19 mmhg, *p* = 0.07).

Reproducibility was good to excellent for all myocardial work parameters, with high ICC and low CV (Supplementary Table 2). Slightly higher variability was observed for MWW.

### LV function at baseline and follow-up

All patients initially had an LVEF less than 50% at inclusion, whereas at baseline evaluation on the day of PET scan, LVEF was less than 50% in 5 patients. Mean EF at baseline and at the 6-month follow-up was similar (55.8±6.8 vs 57.5 ± 7.3%, *p* = 0.3, Table 2). Furthermore, at least 5% EF recovery was detected in 12 patients (40%, functional recovery group). EF at the baseline was comparable between the functional recovery group and the no recovery group (53±6.4 vs 57.7 ± 6.7%, *p* = 0.07), whereas EF was higher in the functional recovery group at the 6-month follow-up (60.9±5.8 vs 55.2 ± 7.4, *p* = 0.03). The mean EDV and ESV were similar at baseline and at the 6-month follow-up (94.7±27.7 vs 93.6 ± 30.1 ml, *p* = 0.7, and 42.7 ± 17.1 vs 40.8 ± 19.1, *p* = 0.4, respectively). Similar to EF, GLS remained similar between baseline and the 6-month follow-up echocardiography (14.9±4.6 vs 15.4 ± 3.9, *p* = 0.3, Table 2). In the myocardial AAR, regional LS increased at the 6-month follow-up as compared to baseline, whereas in the remote area there were no differences between baseline and follow-up LS (Table 2).

**Table 2:** Changes in echocardiographic variables between baseline and 6- month follow-up

	Baseline	Follow-up	*p*-value
Global variables			
EF (%)	55.84 ± 6.79	57.47 ± 7.3	0.3
GLS (%)	14.9 ± 4.6	15.4 ± 3.9	0.3
MWI (mmHg %)	1317.79 ± 405.09	1711.97 ± 533.75	<0.001
MCW (mmHg %)	1683.1 ± 466	1997.74 ± 574	<0.001
MWW (mmHg %)	213.38 ± 137.45	117.66 ± 96.56	0.002
MWE (%)	89 (82–93)	95 (91- 96)	0.003
Regional variables			
Area at risk			
LS (%)	12.5 ± 9.5*	14.5 ± 9 **	0.005
MWI (mmHg %)	1172.82 ± 483.5*	1552.02 ± 562.41*	<0.001
MCW (mmHg %)	1370.55 ± 453.51*	1745.81 ± 610.69*	<0.001
MWW (mmHg %)	284.62 ± 232.98	158.8 ± 109.71	<0.001
MWE (%)	87.75 (81–95) *	92.2 (84–96) **	0.05
Remote area			
LS (%)	15.71 ± 9.4	17.2 ± 9	0.2
MWI (mmHg %)	1577 ± 509.28	1868.35 ± 518.59	<0.001
MCW (mmHg %)	2018.92 ± 642.16	2224.13 ± 604.45	0.02
MWW (mmHg %)	207.53 ± 206	166.85	0.4
MWE (%)	91(78–97)	95 (91–98)	0.8

EF, ejection fraction; GLS, global longitudinal strain; LS, longitudinal strain; MWI, myocardial work index; MCW, myocardial constructive work; MWW, myocardial wasted work; MWE, myocardial work efficiency. Values are expressed as mean ± SD or median (IQR). *vs. remote area, *p* < 0.001, ** vs remote area, *p* < 0.01. GLS and LS represent absolute values

### Trajectories of global and regional MW indices after AMI

Table 2 shows the echocardiographic MW indices of the study cohort. Global MWI, MCW and MWE significantly increased at the 6-month follow-up compared to baseline, whereas MWW decreased

**Fig. 1:**
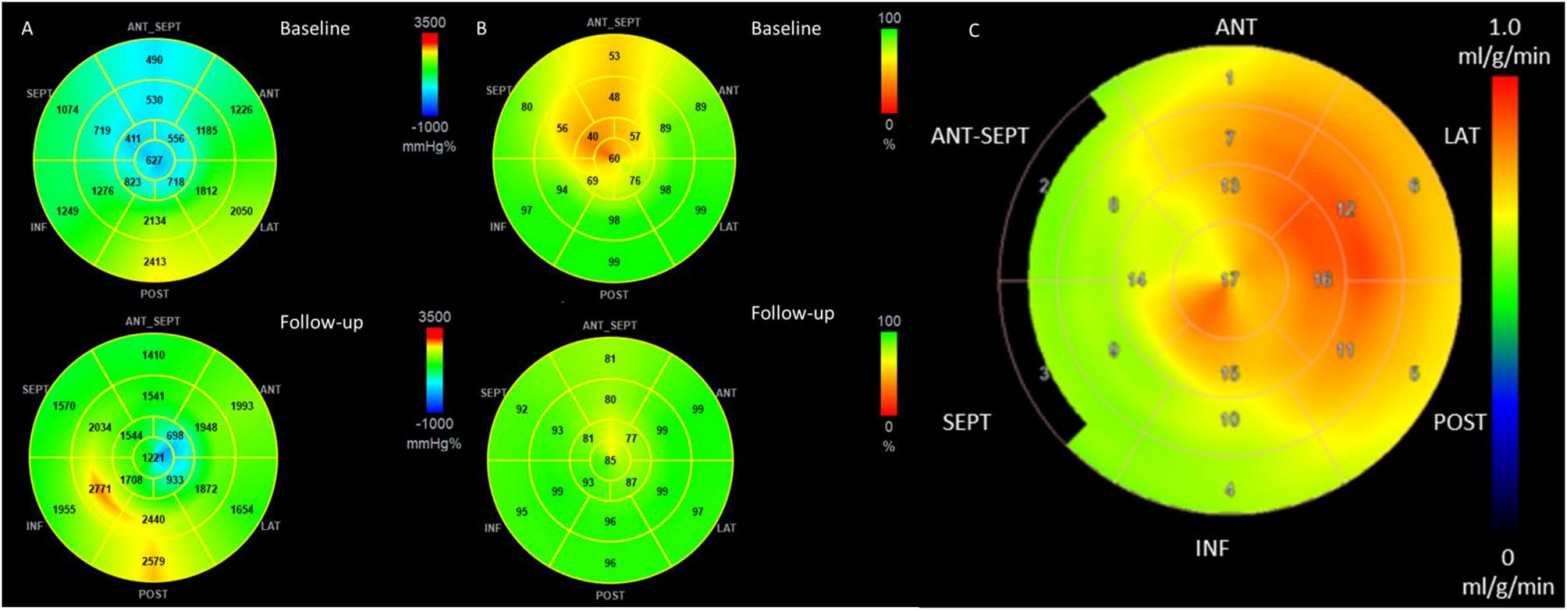
Evaluation of myocardial work indices and perfusion in patient with acute myocardial infarction polar maps showing echocardiographic myocardial work indices (**A** and **B**) and resting myocardial blood flow (**C**) assessed by 15-oxygen water positron emission tomography (PET) in a patient with acute myocardial infarction caused by occlusion of the proximal left anterior descending coronary artery. Myocardial work index (**A**) and myocardial work efficiency (**B**) Are reduced in the anteroseptal region at baseline (upper panels) and show partial recovery at the 6-month follow-up (lower panels). Myocardial blood flow is reduced in the anteroseptal region. Please note different orientation of echocardiographic and PET polar maps

In the AAR, MWI, MCW and MWE were significantly lower than in the remote area both at baseline (all *p* < 0.001) and at the 6-month follow-up (all *p* < 0.01). In the AAR, MWI and MCW significantly increased at the 6-month follow-up, whereas MWW decreased. The increase in MWE was borderline significant (*p* = 0.05). In the remote area, MWI and MCW increased, but other myocardial work indices remained unchanged. An illustrative example demonstrating reduced myocardial work indices and perfusion in the anteroseptal region with partial recovery at follow-up is shown in Fig. 1.

### Myocardial work indices and LV functional recovery

**Fig. 2:**
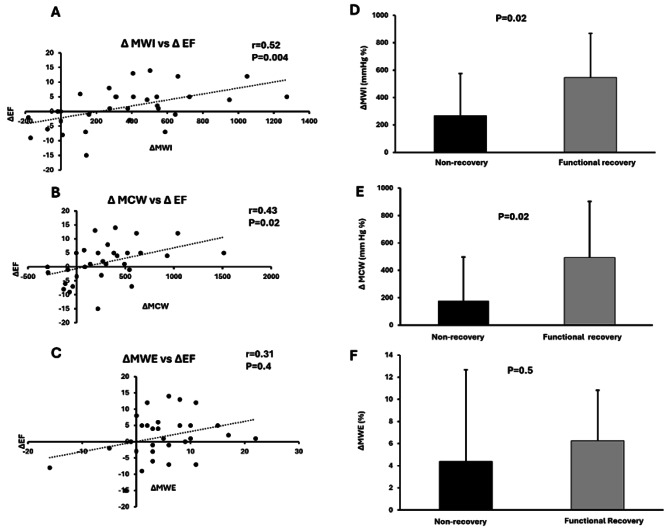
Association between changes in myocardial work indices and left ventricular ejection fraction (LVEF) recovery. (**A**–**C**) scatter plots illustrating correlations between changes in myocardial work index (ΔMWI), constructive myocardial work (ΔMCW), and myocardial work efficiency (ΔMWE) with changes in LVEF (ΔEF). Pearson’s correlation coefficients (**r**) and *p*-values are shown. (**D**–**F**) Bar graphs comparing ΔMWI, ΔMCW, and ΔMWE between patients with functional recovery (grey) and those without recovery (black). Data are presented as mean ± standard deviation

Baseline MWI, MCW, MWW and MWE were similar between functional recovery and no recovery groups. The change in global MWI and MCW correlated significantly with the change in EF and GLS (*r* = 0.52, *p* = 0.004, and *r* = 0.43, *p* = 0.02, respectively) from baseline to the 6-month follow-up (Fig. 2A and B). Furthermore, global MWI and MCW increased significantly more in the functional recovery group than in the no recovery group (Δ MWI 546.8, 95% CI [333.1 to 760.5] vs 266.4, 95% CI [108.4 - 424.5] mm Hg %, *p* = 0.02, and Δ MCW 493.1, 95% CI [220.8 - 765.3] vs 175.1, 95% CI [10.5- 339.6] mm Hg %, *p* = 0.02, respectively) (Fig. 2D and E). However, changes in global MWE and MWW were neither correlated with changes in EF (*r* = 0.31, *p* = 0.4 and *r* = −0.27, *p* = 0.5, respectively) nor were significantly different between the functional recovery and no recovery groups (Δ MWE 5.2, 95% CI [−10.9 to 0.5] vs 7.7, 95% CI [−13.5 to −1.9] %, *p* = 0.4 and Δ MWW −96.7, 95% CI [12.18 to 181.16] vs −95.8, 95% CI [−10.92 to 202.42], *p* = 0.9, respectively) (Fig. 2C and F).

### Determinants of myocardial work indices

**Fig. 3:**
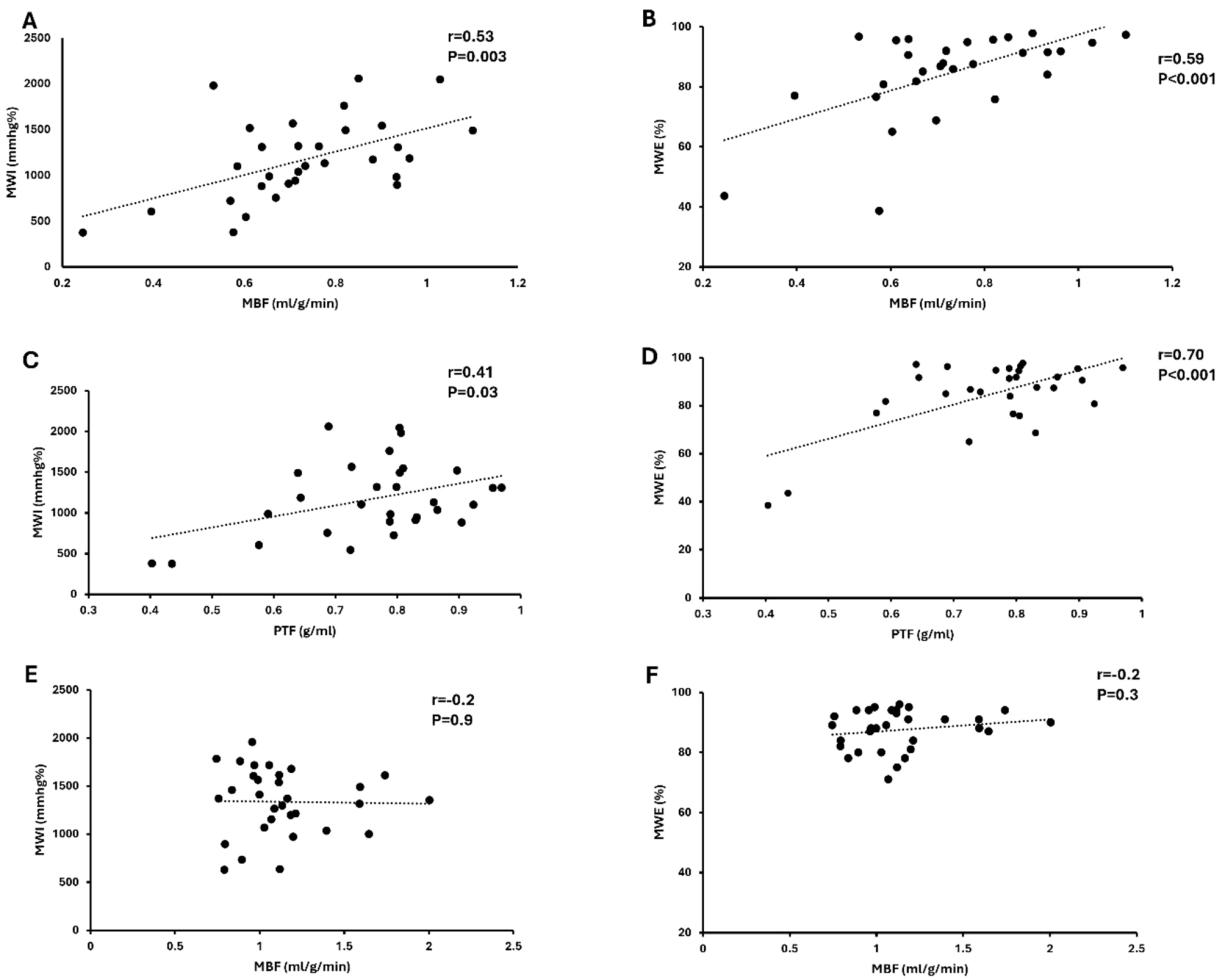
Correlations between myocardial work index (MWI), myocardial work efficiency (MWE), myocardial blood flow (MBF) and perfusable tissue fraction (PTF) in the area at risk. Scatter plots demonstrating the correlation between myocardial work indices and PET-derived perfusion parameters. (**A**) Myocardial work index (MWI) vs. myocardial blood flow (MBF) in the area at risk, (**B**) Myocardial work efficiency (MWE) vs. MBF in the area at risk, (**C**) MWI vs. perfusable tissue fraction (PTF) in the area at risk, (**D**) MWE vs. PTF in the area at risk, (**E**) global MWI vs. corrected global MBF, (**F**) global MWE vs. corrected global MBF

The mean global corrected resting MBF and PTF at rest were 1.14 ± 0.30 mL/g/min and 0.73 ± 0.11 g/ml, respectively. Mean resting MBF was 0.87 ± 0.20 mL/g/min in the AAR and 0.73 ± 0.18 mL/g/min in the remote area (*p* = 0.008). As shown in Fig. 3, global MWI or MWE were not correlated with global MBF. In the AAR, however, MWI and MWE correlated with regional MBF (*r* = 0.53, *p* = 0.003, and *r* = 0.59, *p* < 0.001) and PTF (*r* = 0.41, *p* = 0.03, and *r* = 0.65, *p* < 0.001, Fig. 3).

Global MWI or MWE were inversely correlated with peak levels of troponin and NT-proBNP at baseline (Table 3). MWI and MWE correlated with LV EF and GLS both at baseline and at the 6-month follow-up (Table 3 and Supplementary Table 3). Furthermore, at baseline, MWI showed an inverse correlation with ESV. At the 6-month follow-up, both MWI and MWE were inversely correlated with ESV. Neither MWI nor MWE showed significant correlations with EDV (Table 3).

**Table 3:** Correlations between global MWI or MWE and EF, EDV, ESV, MBF, TnT, and NT-proBNP, at baseline or 6-month follow-up

	MWI		MWE	
	r	p-value	r	p-value
**Baseline**				
EF	0.66	<0.001	0.52	0.003
EDV	−0.31	0.6	−0.07	0.9
ESV	−0.49	0.005	−0.21	0.6
TnT	−0.49	0.007	−0.45	0.01
NT-proBNP	−0.6	<0.001	−0.38	0.04
**Follow-up**				
EF	0.68	<0.001	0.75	<0.001
EDV	−0.2	0.4	−0.3	0.5
ESV	−0.44	0.01	−0.51	0.004

MWI, myocardial work index; MWE, myocardial work efficiency; EF, ejection fraction; EDV, end-diastolic volume; ESV, end-systolic volume; MBF, myocardial blood flow; TnT, Peak troponin T; NT-proBNP, Peak NT-proBNP

### Myocardial work indices and myocardial viability

Table 4 and Supplementary Figure 2 summarize regional MW indices and LS according to myocardial viability based on PTF. At baseline, LS, MWI, MCW, and MWE were higher in viable segments (416 segments, PTF ≥ 0.60 g/ml) than non-viable segments (45 segments, PTF < 0.60 g/ml), whereas MWW was similar. Furthermore, MWI, MCW, and MWE predicted segmental myocardial viability (Supplementary table 4). MWI ≥ 945 mmHg% and MCW ≥ 1202 mmHg% were the best cut-off values for prediction of segmental myocardial viability with AUCs of 0.708 and 0.697, respectively (both *p* < 0.001), while MWE ≥ 83% had a lower AUC of 0.614 (*p* < 0.02) (Supplementary Figure 3).

**Table 4:** Comparison of regional longitudinal strain and myocardial work indices between viable and non-viable myocardial segments at baseline and at 6-month follow-up

Parameter	Baseline			6 months		
	Viable (n = 416)	Non-viable (n = 45)	p-value	Viable (n = 416)	Non-viable (n = 45)	p-value
Regional LS (%)	15 ± 5.3	8.6 ± 9.6	<0.001	15.6 ± 6.5	9.7 ± 7.3	<0.001
Regional MWI (mmHg)	1346.7 ± 553.2	974.4 ± 740.9	<0.001	1716.4 ± 630.9*	1075.2 ± 661.3	<0.001
Regional MCW (mmHg)	1714.9 ± 657.1	1276.9 ± 855.8	<0.001	2023.0 ± 716.5*	1333.5 ± 752.0	<0.001
Regional MWW (mmHg)	146 (65–113)	155 (93–281)	0.7	82 (56–212) *	180 (90–345)	<0.001
Regional MWE (%)	90 (82–95)	87 (73–93)	0.01	95 (89–98) *	86 (73–94)	<0.001

LS, longitudinal strain; MWI, myocardial work index; MCW, myocardial constructive work; MWW, myocardial wasted work; MWE, myocardial work efficiency. Values are expressed as mean ± SD or median (IQR). *p*-values indicate comparisons between viable and non-viable myocardial segments at each time point. *p < 0.05 for within-group change from baseline to 6 months

In the viable segments, MWI, MCW, and MWE increased and MWW decreased from baseline at the 6-month follow-up. In contrast, in non-viable segments all MW indices were similar at baseline and the 6-month follow-up and remained lower (MWI, MCW and MWE) or higher (MWW) than in the viable segments (Table 4).

## Discussion

The main findings of our study can be summarized as follows: The global LV echocardiographic myocardial work indices including parameters of myocardial work and work efficiency improved from 1 week to 6 months after ST-elevation AMI. The increase in global myocardial work paralleled an increase in LVEF. Early after AMI, decreased myocardial work efficiency was associated with low LVEF, large MI size and increased NT-proBNP, whereas there was no association to global MBF. Furthermore, preserved myocardial work indices were associated with myocardial viability early after AMI. Over the 6-month follow-up, all myocardial work indices demonstrated improvement in viable segments, whereas non-viable segments showed no significant changes. These findings indicate that myocardial work and work efficiency are potential markers of viable myocardium and functional recovery after AMI.

Previous studies have shown that myocardial work indices are potentially useful tools for evaluating LV function in various loading conditions by examining LV longitudinal deformation in the context of the corresponding pressure [13]. Hedwig et al. reported that low global MWI was associated with impaired ejection fraction, LV remodelling, low exercise capacity, and elevated NT-pro-BNP in patients with chronic heart failure. [14]. Edwards et al. evaluated myocardial work indices in patients with suspected obstructive CAD with EF ≥ 55% and no resting regional wall motion abnormalities [15]. The study showed that all global myocardial work indices were impaired in patients with obstructive CAD, and reduced global MWI was superior in predicting the presence of obstructive CAD as compared to other echocardiographic parameters, including GLS and EF. Another study showed that regional MWI and MCW were impaired in the LV segments perfused by stenotic coronary arteries associated with reduced fractional flow reserve in patients with chronic CAD [16]. Furthermore, regional MWI and MCW were improved after revascularization [16]. Moreover, Boe et al. found that regional MWI provided incremental value for the detection of acute coronary artery occlusion in non-ST-elevation MI [3]. In line with these studies, we found that MW indices were significantly reduced in the ischemic AAR compared to the remote myocardium at both baseline and follow-up. We also found that global MWI and MWE showed a significant positive correlation with EF and GLS both at baseline and 6-month follow-up. These findings indicate that evaluation of myocardial work indices may be useful as markers of ischemic myocardial injury and LV function after AMI. [16]

Post-AMI heart failure is a clinical problem, and the prediction of LV functional recovery is challenging [1, 17, 18]. In our study, we found that myocardial work indices were associated with established heart failure predictors after AMI, including increased levels of TnT, and NT-proBNP, reduced LVEF and increased LV ESV. [1] Our results are consistent with recent studies, showing that TnT and BNP levels were inversely correlated with global myocardial work indices [14, 19] and that myocardial work indices were impaired in patients with left ventricular remodelling after MI [20] Interestingly, impaired myocardial work indices early after MI have been found as predictors of left ventricular remodelling, whereas preserved indices predict recovery of LV function [20]. In order to study this further, we evaluated associations between myocardial work indices, myocardial viability and myocardial blood flow.

Myocardial viability at the segment level is an important determinant of functional outcome after revascularisation in patients with STEMI [21]. A strong correlation between MWI and regional glucose metabolism measured with 2-deoxy-2-[^18^F] fluoroglucose ([^18^F] FDG) PET has been found, suggesting its potential as a marker of myocardial viability [22]. El Mahdiui et al. [23] assessed myocardial work indices and myocardial viability determined by late gadolinium enhancement cardiac magnetic resonance in STEMI patients at variable time points after ST-elevation AMI. They found that MCW and MWE were significantly lower in segments with transmural infarction. In line with this, we found that MWI, MCW, and MWE were significantly reduced in non-viable myocardial segments as compared to viable segments at baseline, which persisted after six months. We evaluated viability using [^15^O] water PET and PTF, which is an established index for assessing myocardial viability aligning with histology [9], [^18^F] FDG PET [12, 24] or LGE CMR [25]. While CMR is widely considered the reference standard for comprehensive myocardial tissue characterization, including assessment of infarct size, edema, and microvascular obstruction [21], [15O]-water PET enables quantitative assessment of MBF and PTF. An important advantage of PET is the ability to simultaneously assess myocardial perfusion and viability in a fully quantitative manner at the segmental level, which is particularly relevant for comparison with regional myocardial work indices. However, PET is less widely available than CMR, which may limit its broader clinical applicability. In addition, PET does not provide detailed tissue characterization, such as assessment of myocardial edema, and fibrosis.

Based on these findings, we further evaluated the diagnostic performance of myocardial work indices for the identification of viable myocardium. The ROC results demonstrated good diagnostic performance for viability assessment, with MWI (≥945 mmHg %) and CMW (≥1202 mmHg %) showing AUCs of 0.708 and 0.697, respectively. Notably, these values are comparable to previous study, which reported AUCs of 0.7 ± 0.02 for MWI and 0.73 ± 0.02 for CMW [19] to predict functional improvement 3 months after AMI In contrast, while MWE showed a stronger association with viability in logistic regression (OR = 1.029, *p* = 0.004), its lower AUC (0.614, *p* < 0.02) suggests limited diagnostic accuracy. This finding is also consistent with prior research, where MWE had a slightly higher AUC (0.64 ± 0.02) but remained less predictive than MWI and CMW [19]. The discrepancy between MWE’s strong association in regression and its weaker ROC performance suggests that MWE may reflect preserved myocardial function rather than viability [26]. The former may remain relatively preserved even in segments with reduced viability and MWE values include MWW in their calculation [19]. Furthermore, we found that MW indices improved only in viable regions from 7 days to the 6-month follow-up. Thus, our findings support earlier results suggesting that MW indices can be used as potential indicators of segmental myocardial viability and functional recovery after AMI [19]

We also assessed the relationship between the MW indices and myocardial perfusion by PET. Because resting MBF is heavily dependent on oxygen consumption [27], we hypothesized that myocardial work indices correlate with MBF. Indeed, previous studies found a correlation between MW indices and peak oxygen consumption in patients with heart failure [14, 28]. However, we found that there was no correlation between global MW indices and MBF after AMI. This may be related to interplay between the duration of post-ischemic functional impairment in reperfused viable segments and reduced MBF in non-viable segments in the AAR. In line with this, we found that regional MWI and MWE values in the AAR inversely correlated with regional MBF and PTF.

## Limitations

This was a single-centre study with relatively few patients. The relatively wide imaging window (3–14 days) may have influenced myocardial work and PET parameters, as myocardial stunning and microvascular recovery evolve during this period. However, no significant differences were observed between patients imaged ≤ 7 and >7 days after AMI, suggesting a limited impact on the overall findings. The low number of clinical events and the relatively small sample size limit the ability to evaluate the prognostic value of myocardial work indices in this cohort. Larger studies are needed to assess their potential role in predicting clinical outcomes. Our ability to detect associations between the MW indices and changes in LV structure and function may have been limited by the modest degree of changes and a limited number of patients with significant LV remodelling despite a relatively long time from symptom onset to revascularization. For evaluation of the myocardial viability, we used PTF assed by [^15^O] water PET instead of FDG PET. However, PTF has yielded comparable results with the [^18^F] FDG and could be considered an alternative for viability assessment [12, 24]. Finally, Longer-term outcome data were unavailable for this study, so we could not determine whether MWI indices are useful in predicting clinical outcomes after AMI at long-term follow-up.

## Conclusion

Myocardial work indices improve after AMI in parallel with LVEF. In the early post-infarction phase, these indices are associated with established markers of myocardial injury, including infract size, LVEF, and NT-proBNP levels. Although myocardial work indices do not correlate with global resting myocardial blood flow, they are significantly impaired in non-viable myocardial segments and fail to improve over time. These findings highlight the potential of myocardial work indices as non-invasive markers of myocardial injury and functional recovery after AMI.

## Electronic supplementary material

Below is the link to the electronic supplementary material.

Supplementary Material 1

## Data Availability

The datasets used and/or analysed during the current study are available from the corresponding author on reasonable request.
