# Supplementary material for: Trajectories and determinants of echocardiographic myocardial work indices after acute myocardial infarction: a pilot study
Source: Echo Res Pract. 2026 Aug 10;13:29. doi: 10.1186/s44156-026-00131-5 (PMC13455251; doi:10.1186/s44156-026-00131-5)
Supplement: Supplementary file 1 [file 44156_2026_131_MOESM1_ESM.docx]

**Supplementary data**

**Supplementary Tables**

**Supplementary Table 1.** Baseline echocardiographic and PET parameters according to timing of baseline imaging

| Parameter | ≤7 Days (n=15) | >7 Days (n=16) | p-value |
| --- | --- | --- | --- |
| Echocardiography |  |  |  |
| EF (%) | 54.33±11.6 | 57.25±12.31 | 0.2 |
| GLS (%) | 14.13 ± 7.98 | 14.31 ± 7.64 | 0.9 |
| MWI (mmHg%) | 1354 (1050-1417) | 1390 (1247- 1544) | 0.8 |
| MCW (mmHg%) | 1652 (1342-2045) | 1733(1426- 1897) | 0.6 |
| MWW (mmHg%) | 197 (128-293) | 132 (91-240) | 0.3 |
| MWE (%) | 88 (83-91) | 91 (86-94) | 0.2 |
| PET |  |  |  |
| MBF (mL/g/min) | 0.8586 ± 0.354 | 0.8311 ± 0.344 | 0.7 |
| PTF (g/mL) | 0.7315 ± 0.357 | 0.7287 ± 0.357 | 0.9 |

EF, ejection fraction; GLS, global longitudinal strain; MWI, myocardial work index; MCW, myocardial constructive work; MWW, myocardial wasted work; MWE, myocardial work efficiency; MBF, myocardial blood flow; PTF, perfusable tissue fraction. The data are expressed as mean ± SD or median (interquartile range).

**Supplementary Table 2.** Intra- and inter-observer variability of myocardial work and strain parameters assessed using intraclass correlation coefficients (ICC) and coefficients of variation (CV) in 6 patients.

| Parameter | Intra-observer | | | Inter-observer | |
| --- | --- | --- | --- | --- | --- |
|  | **ICC** | **CV (%)** | **ICC** | | **CV (%)** |
| GLS | 0.94 | 2.4 | 0.99 | | 4.5 |
| MWI | 0.93 | 2.7 | 0.96 | | 3.4 |
| MCW | 0.96 | 8.3 | 0.97 | | 3.7 |
| MWW | 0.86 | 13.8 | 0.95 | | 12.7 |
| MWE | 0.97 | 2.1 | 0.98 | | 1.7 |

GLS, global longitudinal strain; MWI, myocardial work index; MCW, myocardial constructive work; MWW, myocardial wasted work; MWE, myocardial work efficiency.

**Supplementary Table 3.** Correlations between global longitudinal strain and myocardial work indices.

| Correlation | Baseline | | 6-month follow-up | |
| --- | --- | --- | --- | --- |
|  | **r** | **p-value** | **r** | **p-value** |
| MWI | 0.88 | <0.001 | 0.8 | <0.001 |
| MCW | 0.83 | <0.001 | 0.81 | <0.001 |
| MWW | -0.4 | 0.02 | -0.5 | 0.005 |
| MWE | 0.71 | <0.001 | 0.77 | <0.001 |

MWI, myocardial work index; MCW, myocardial constructive work; MWW, myocardial wasted work; MWE, myocardial work efficiency.

**Supplementary Table 4.** Binary logistic regression analysis for myocardial work indices in predicting myocardial viability determined by PTF.

| Variable | Odds Ratio | 95% CI | p-value |
| --- | --- | --- | --- |
| MWI | 1.0011 | 1.0005 – 1.0017 | <0.001 |
| CMW | 1.0012 | 1.0006 – 1.0019 | <0.001 |
| MWW | 1.25 | 0.8000-1.8304 | 0.25 |
| MWE | 1.029 | 1.0093 – 1.0478 | 0.004 |

MWI, myocardial work index; MCW, myocardial constructive work; MWW, myocardial wasted work; MWE, myocardial work efficiency.

**Supplementary Figures**

**Supplementary Figure 1**. Distribution of time from acute myocardial infarction to baseline imaging.

**Supplementary Figure 2.** Changes in Myocardial Work Indices in Viable and Non-Viable Myocardial Segments from Baseline to 6-Month Follow-up

Panels (A–D) compare myocardial work indices between non-viable (blue) and viable (red) myocardial segments at baseline and 6-month follow-up. Myocardial Work Index (A), Constructive Myocardial Work (B), Myocardial Wasted Work (C), Myocardial Work Efficiency (D). * vs. Baseline p≥0.05, ** vs. baseline p<0.001

**Supplementary Figure 3.** ROC curves of myocardial work indices to predict the viability.

(Blue) MCW at cutoff ≥ 1202 mmHg % Sensitivity= 80%, Specificity= 65%, AUC, 0.697, 95% CI (0.587-0.805), P<0.001

(Green) MWI, at cutoff ≥ 945 mmHg % Sensitivity= 77%, Specificity= 70%, AUC, 0.708, 95% CI (0.604-0.813), P<0.001

(Red) MWE, at cutoff ≥ 83 % Sensitivity= 75%, Specificity= 52%, AUC, 0.614, 95% CI (0.524-0.704), P<0.02
